# Antenatal care providers’ attitudes and beliefs towards maternal vaccination in Kenya

**DOI:** 10.12688/gatesopenres.13091.1

**Published:** 2020-02-05

**Authors:** Subhash Chander, Ines Gonzalez-Casanova, Sandra S. Chaves, Nancy A. Otieno, Marc-Alain Widdowson, Jennifer Verani, Paula Frew, Andrew Wilson, Saad B. Omer, Fauzia Malik

**Affiliations:** 1 Department of Global Health, Emory University, Atlanta, GA, 30322, USA; 2 Influenza Program, Kenya Center for Disease Control and Prevention, Nairobi, Kenya; 3 Center for Global Health Research, Kenya Medical Research Institute, Kisumu, Kenya; 4 Division of Global Health Protection, Kenya Center for Disease Control, Nairobi, Kenya; 5 Population Health & Health Equity Initiative, University of Nevada Las Vegas, Las Vegas, NV, USA; 6 School of Public Health, University of Nevada Las Vegas, Las Vegas, NV, USA

**Keywords:** Antenatal care providers, healthcare providers, vaccine acceptance, maternal vaccination, knowledge, attitudes and beliefs

## Abstract

**Background:** Maternal immunization is known to be one of the best strategies to protect both mothers and their infants from infectious diseases. Studies have shown that healthcare providers play a critical role in implementation of maternal immunization. However, little is known about providers’ attitudes and beliefs towards vaccination that can influence their vaccine recommendations, specifically in low to middle income countries (LMIC).

**Methods: **A self-administrated knowledge, attitude and behavior (KAB) survey was provided to 150 antenatal care providers across four different regions (Nairobi, Mombasa, Marsabit, and Siaya counties) of Kenya. The research staff visited the 150 clinics and hospitals and distributed a quantitative KAB survey.

**Results: **Nearly all of the antenatal care providers (99%) recommended tetanus maternal vaccination. Similarly, 99% of the providers agreed that they would agree to provide additional vaccinations for pregnant women and reported that they always advise their patients to get vaccinated. Between 80 and 90% of the providers reported that religious beliefs, ethnicity, cultural background and political leaders do not affect their attitude or beliefs towards recommending vaccines.

**Conclusions: **Considering the positive responses of healthcare providers towards vaccine acceptance and recommendation, these results highlight an opportunity to work in partnership with these providers to improve coverage of maternal vaccination and to introduce additional vaccines (such as influenza). In order to achieve this, logistical barriers that have affected the coverage of the currently recommended vaccines, should be addressed as part of this partnership.

## Introduction

Young infants remain highly vulnerable to infectious diseases ^1^, partially because vaccination is not feasible or effective for most diseases during the first months of life ^1^. Maternal immunization has the potential to yield protection for both the mother and their infant ^2^. Clinical studies have demonstrated protection of infants against various infectious diseases such as pertussis and influenza through the placental transfer of antibodies from vaccinated mothers ^3, 4^. Hence, the importance of promoting maternal immunization, especially in settings where the risk of infection during pregnancy and early infancy is high.

Despite proven advantages and significant progress in maternal immunization worldwide, many countries in Africa, including Kenya, recommend only tetanus- diphtheria (Td) vaccination for pregnant women, and coverage remains suboptimal in some regions ^5^. Some of the determinants of low vaccine uptake in the African region include living in urban or peri-urban areas; few dedicated economic and human resources; lack of sufficient vaccines due to demand and supply inconsistencies and barriers to vaccine acceptance by pregnant women and their communities ^5– 7^. Healthcare providers can play a key role in overcoming these barriers. Studies in the United States have shown that women who had discussions about vaccine benefits with their antenatal care providers were more likely to accept vaccine during pregnancy ^8^.

Pregnant women have shown that they rely and trust healthcare providers for immunization related information ^2^. A couple of studies in Asia and Africa have shown that healthcare provider recommendation not only improve vaccine acceptance in pregnant women but can also motivate their male-partners to accept maternal vaccination. This patient-provider relationship has been seen particularly important in low-to-middle income countries (LMIC) ^9, 10^. Providers’ attitudes and beliefs towards vaccination have also been shown to influence vaccine recommendations for pregnant women ^11^. Despite high morbidity and mortality of vaccine preventable disease in LMIC such as Kenya ^12^, most of the research assessing the knowledge, attitudes, and beliefs of health providers towards maternal immunization has been conducted in high- income settings. The objective of this study was to assess attitudes, beliefs and characteristics of antenatal care providers towards maternal vaccination in Kenya.

## Methods

### Study design

Data for this analysis are part of a larger study aimed at identifying determinants of maternal vaccine acceptance in Kenya, which was conducted between June 2016 and August 2018 *.* The study was conducted by Emory University, in collaboration with the Centers for Disease Control and Prevention (CDC) and the Kenya Medical Research Institute (KEMRI). Approval for the study was obtained from Emory University’s [IRB00089673] and KEMRI’s Institutional Review Boards [SSC 3292]. Written informed consent was obtained from participants before enrolling in the study.

### Study population

The study population included 150 antenatal care providers working in antenatal care clinics and hospitals, from primary care to referral settings, in four different areas in Kenya (Nairobi, Mombasa, Marsabit, and Siaya counties). The sample size was calculated in order to estimate correlations between predictors and ANC responses based on a conservative distribution of 50% for response variables, assuming 80% power and an alpha of 0.05. The inclusion criteria for participants were being employed in a clinic or hospital in the target sites as an ANC provider and providing services to pregnant women. The recruitment sites varied from small clinics to large hospitals with patient population ranges between tens to hundreds of women. The study sites were selected to represent the geographic diversity of Kenya and based on the study team ability to access them: Nairobi is the capital and largest city of Kenya; Mombasa is a coastal city with a majority Muslim population; Marsabit is a remote region with low population density and nomadic groups; and Siaya represents western Kenyan rural region.

### Data collection

The research staff visited the 150 clinics and hospitals and distributed a quantitative knowledge, attitude and behavior (KAB) survey to the antenatal care (ANC) providers (see extended data for questionnaire ^13^). The survey was developed based on information collected in the qualitative phase of the study, which included 111 semi-structured interviews with ANC providers ^14^ and pilot tested by the study team in all sites. Participants were recruited both as a convenience sample from study facilities and referral through the healthcare workers and colleagues. The self-administered KAB included questions on vaccine-preventable diseases (including burden and perceived risk), vaccine effectiveness, vaccine safety, vaccination norms, prior experience with vaccination (either for themselves, their children, their patients, etc.), positive and negative motivations to vaccinate, and values around vaccination. The survey also collected socio-demographic information. All the questionnaires were translated into the local languages, including Luo, Kikyo, Luhya, Kamba, Swahili, Mijikenda, Taita, Borana, Rendile, Burji and Somali. For the purpose of analysis, the questionnaires were translated back to English.

### Data analysis

Demographic variables were categorized as follows: age, education and marital status were dichotomized (<30 vs. ≥30 years; college or less vs. more than college; and single vs. married/cohabitation) respectively. Religion was divided into four categories: catholic, protestant, Muslim and traditional African churches/traditional religion/others.

To get an aggregate of positive, neutral or negative responses, we collapsed the five item Likert scale into three. Strongly agree and agree were summarized as agree and strongly disagree and disagree were summarized as disagree. 

Descriptive statistics (means and standard deviations, proportions) were summarized for all the variables and survey questions. using SAS, version 9.4 (SAS Institute, Cary, NC).

## Results

A total of 150 participants were included in this study (see underlying data ^13^). Most of the participants were female (77.3%), nurses (89.3%) and over 30 years of age (67.3%) ( Table 1).

**Table 1.** Demographic information and characteristics of antenatal care (ANC) provider.

*Characteristic*	n	%
*Female*	116	77.3
***Age***		
*18 to 29 years*	49	32.7
*Over 30 years*	101	67.3
***Level of Education***		
*College or less than college*	130	86.7
*More than college education*	20	13.3
***Religion***		
*Catholic*	53	35.3
*Protestant*	67	44.7
*Traditional African churches/traditional religion/others*	7	4.7
*Muslims*	23	15.3
***Healthcare Staff***		
*Nursing*	134	89.3
*General/Internal Medicine*	7	4.7
*Pediatrics*	3	2.0
*Obstetrics/Gynecology*	1	0.7
*Surgery*	2	1.3
***Marital Status***		
*Single/Divorced/Separated/Widow/Widower*	45	30.9
*Married/Cohabitation*	105	69.1
***Mother Tongue***		
*Luo*	49	32.7
*Kikyu*	18	12.0
*Luhya*	11	7.3
*Kamba*	14	9.3
*Swahili*	6	4.0
*Mijikenda*	3	2.0
*Taita*	5	3.3
*Borana\Rendile\Burji\Somali*	28	18.7
*Other*	16	10.7

Nearly all of the ANC providers had positive attitudes towards maternal vaccination in general (when no vaccine was specified), agreeing that vaccines are one of the safest strategies to protect both mother and newborns from diseases, and can be administered even when they are suffering from chronic conditions such as HIV. Nearly 80% of the providers agreed that influenza is a matter of concern in pregnant women. Approximately 97% of the providers agreed that Td vaccine is effective and should be administered in pregnancy ( Table 2).

**Table 2.** Antenatal care (ANC) provider knowledge, attitudes and beliefs on vaccination.

*n (%)*	Agree	Neutral	Disagree
*I recommend to all my pregnant patients that they should be vaccinated.*	149(99.3)	0(0.0)	1(0.7)
*I think there should be more recommended vaccines for pregnant women*	149(99.3)	1(0.7)	0(0.0)
*Vaccines are necessary for pregnant women for their own protection from diseases.*	143(95.3)	6(4.0)	1(0.7)
*Vaccines are necessary for pregnant women for protection of unborn children from* *diseases.*	150(100)	0(0.0)	0(0.0)
*Vaccines are safe for use in pregnancy.*	142(94.7)	3(3.0)	5(3.3)
*I am concerned that vaccines may weaken the immune system of pregnant women*	9(6)	2(1.3)	139(92.7)
*I am concerned that too many vaccines could bring complication to pregnant woman’s* *immune system*	39(26)	7(4.7)	104(69.3)
*Vaccines are getting better and safer as a result of medical research*	147(98.0)	3(2.0)	0(0.0)
*Vaccinating pregnant women can cause infertility*	1(0.7)	1(0.7)	148(98.7)
*Vaccinating pregnant women can cause disability.*	5(3.3)	5(3.3)	140(93.3)
*Vaccines cause miscarriage or still birth in pregnant women*	4(2.7)	2(1.3)	144(96.0)
*Vaccines are safe for pregnant women living with HIV*	142(94.7)	4(2.7)	4(2.7)
*Vaccines are safe for pregnant women with anemia*	139(92.7)	7(4.7)	4(2.7)
*Tetanus vaccine is effective when used in pregnancy*	146(97.3)	2(1.3)	2(1.3)
*Tetanus vaccine should be given to pregnant women.*	137(91.3)	6(4)	7(4.7)
*The flu is not a concern for pregnant women*	25(16.7)	5(3.3)	120(80.0)
*Do you think the flu vaccine is risky when provided in pregnancy?*	14(9.3)	44(29.3)	92(61.3)
*Is it safe to vaccinate pregnant women during the first trimester of pregnancy?*	81(54)	2(1.3)	67(44.7)
*Is it safe to vaccinate pregnant women during the second trimester of pregnancy?*	147(98)	1(0.7)	2(1.3)
*Is it safe to vaccinate pregnant women during the third trimester of pregnancy?*	118(78.7)	6(4)	26(17.3)

Providers responded that myths and misconceptions about vaccines in the society did not affect their decisions related to maternal vaccination. A majority also expressed that political leaders do not influence provider’s decision to accept vaccines. Similarly, most participants disagreed that ethnic/cultural background or religious beliefs influenced their attitudes or beliefs towards vaccination. ( Table 3).

**Table 3.** Antenatal care (ANC) provider religious cultural and political belief on vaccination.

*Religious Belief*	Agree	Neutral	Disagree
*My religious affiliation makes it difficult for me to accept vaccines while* *pregnant/my wife is pregnant*	1(0.9)	6(5.2)	109(94)
*My religious affiliation makes it difficult for me to accept vaccines for my children*	0(0)	5(4.7)	102(95.3)
***Ethnicity***			
*My ethnicity makes it difficult for me to accept vaccine while pregnant/my wife is* *pregnant.*	1(0.7)	2(1.5)	131(97.8)
*My ethnicity makes it difficult for me to accept vaccine for my children.*	0(0)	1(0.9)	106(99.1)
***Cultural practices***			
*Some cultural practices prevent me from receiving (allowing my wife to receive) vaccine* *while pregnant.*	75(56)	2(1.5)	57(42.5)
*Despite vaccine refusal by my spouse/father to my child, I would still accept vaccines* *while pregnant*	92(97.9)	9(0.0)	2(2.1)
*Myths can influence me against vaccinations*	146(97.3)	1(0.7)	3(2.0)
*Misconceptions can influence me against vaccinations.*	146(97.3)	0(0.0)	4(2.7)
*Friends encourage me to take up vaccinations.*	126 (84)	9(6.0)	15(10)
*Family members encourage me to take up vaccinations*	121(80.7)	13(8.7)	16(10.7)
***Political Influences***			
*Do opinion leaders influence you against vaccinations?*	6(4)	3(2)	141 (94)
*Do political leaders influence you against vaccinations?*	12(8)	0(0)	138 (92)

Educational resources to guide women about vaccines (92.7%) and supply of vaccines by government sector (87.3%) were reported to be accessible in enough quantity ( Table 4). However, logistical (66%) and human resources (52.7%) were reported to be less available for vaccine delivery. Furthermore, 78% believed that pregnant women take all the scheduled vaccines even when they migrate to new places. In addition, they feel that their patients trusted their suggestions and information about vaccine recommendation.

**Table 4.** Antenatal care (ANC) providers beliefs about availability of supplies.

	Always	*Sometimes*	*Never*
*I have enough vaccine related provider focused educational resources to use.*	96(64.0)	*43(28.7)*	*11(7.3)*
*Does maternal vaccines are easily accessible and available*	137(91.3)	12(8.0)	1(0.7)
*Do you get regular training to get updated information for vaccine?*	64(42.7)	34(22.7)	52(34.7)
*I have enough vaccine educational resources to provide to pregnant mothers*	*139(92.7)*	*6(4.0)*	*5(3.3)*
*I have enough logistical resources to deliver vaccines to pregnant women*	*99(66.0)*	*28(18.7)*	*23(15.3)*
*We have enough human resources to deliver vaccines to pregnant women*	*79(52.7)*	*53(35.3)*	*18(12)*
*It is easy for health facilities to get vaccine supplies from the government*	*131(87.3)*	*15(10.0)*	*4(2.7)*
*Maternal vaccines are easily accessible and available*	*137(91.3)*	*12(8.0)*	*1(0.7)*
*I feel that I have enough information to confidently discuss vaccines with my pregnant* *patients*	*138(92.0)*	*11(7.3)*	*1(0.7)*
*I give pregnant women enough time to review the vaccination information I offer before* *they make a decision whether to refuse or accept.*	*101(67.3)*	*23(15.3)*	*26(17.3)*
*Women trust the vaccine related information that we give them*	*121 (80.7)*	*29 (19.3)*	*0 (0.0)*
*We are updated regularly on vaccination process/information through trainings*	*64(42.7)*	*34(22.7)*	*52(34.7)*
*Despite cultural affiliations, I am able to change my mind to receive vaccines while* *pregnant, when given the right information*	*113(97.4)*	*1(0.9)*	*2(1.7)*

## Discussion

Results from this study of ANC providers in Kenya highlight important avenues to improve coverage with maternal vaccinations that are currently recommended in the country (currently, only TT), as well as opportunities for the introduction of additional vaccines for pregnant women. First, the providers had favorable attitudes towards vaccine administration and believed that a greater number of vaccines should be recommended to protect both mother and the child from preventable diseases and associated debilitating outcomes. Second, providers reported that religion, myths or political opinions do not influence their attitudes and recommendations around maternal vaccination. Third, almost all providers perceived that women consider them as a trustworthy source of information about vaccinations. Finally, it was reported that healthcare centers were well equipped with educational materials. There was also an uninterrupted supply of vaccines from the government sector.

Providers perception of having adequate vaccine supplies was contradictory to a report of 2011 –2015 from the Kenya Division of Vaccines and Immunization, that cited both demand and supply challenges in vaccine availability ^12^. There have been incidents of depleted vaccine supply some African countries, including Kenya and Tanzania ^15^. One of the reasons behind the divergent results might be that our study included mostly accessible clinics and hospitals located within or near urban areas with good infrastructure. It is also possible that efforts to improve vaccine supplies based on previous assessments have been noticed by the providers.

It is important to note that the perceptions of HCPs around maternal immunization are mostly based on the experience with tetanus vaccines (either TT or Td). Maternal immunization against tetanus has been implemented for decades in Kenya. Similarly, partly due to programs to improve coverage, the TT vaccine is regarded as a safe and effective to prevent childhood tetanus ^16^. While the introduction of other maternal vaccines (e.g. influenza) can benefit from the experience with TT, each new vaccine that is introduced will need to be assessed individually and efforts to promote coverage need to be catered to their specific characteristics. 

Efforts to introduce maternal immunization with influenza in Kenya are ongoing. Influenza virus infection was reported as one of the concerns during pregnancy in Kenya ^17^. Since ANC providers are regarded as a main source of information, this is another opportunity where policymakers and immunization managers can partner with HCP to inform and motivate pregnant women to receive an influenza vaccine once the recommendation is enacted.

Globally, studies have shown the influence of ethnicity and cultural background on acceptance of different vaccines ^18– 20^. An encouraging finding from this study was that providers reported that religion, politics and ethnic background did not negatively impact their attitudes and beliefs towards maternal vaccination.

A limitation of this study is that we only included 4 out of 47 counties in Kenya, however the areas selected in our study represented a diversity of geographic areas (low and high population density, urban and rural). Similarly, most of those surveyed were female nurses of Luo ethnicity. Thus, these results may not be representative of the overall KAB among HCWs in Kenya. This might have contributed to the lack of variation in responses which precluded the analysis of predictors. Even though the questionnaire was especially developed based on qualitative work with our target population and it was piloted with practitioners and health workers from the sites of interest, we did not collect data on validity or reliability. Similarly, we did not collect information on the number of providers that were approached and declined to participate. Other limitations are the potential for socially desirable responses. Finally, as previously discussed, only one vaccine (TT) is currently recommended for pregnant women in Kenya.

Taking into account the positive attitudes of healthcare providers, and their recommendations of introduction of new vaccines, this study supports relying on ANC providers as partners to improve maternal vaccine acceptance in Kenya. Campaigns to improve vaccine acceptance in this setting should be implemented in coordination with providers and leverage their willingness to recommend maternal vaccines. It would also be important to identify the sources of training and information that have facilitated this widespread acceptance of maternal immunization among providers in Kenya, and potentially try to replicate these approaches in similar settings.

## Data availability

### Underlying data

Harvard Dataverse: Replication Data for: Antenatal care providers’ attitudes and beliefs towards maternal vaccination in Kenya. https://doi.org/10.7910/DVN/43PPDD ^13^

This project contains the following underlying data:

- MVAC_ANC_GATES manuscript 12 3 2019.tab (Survey responses and data dictionary)

### Extended data

Harvard Dataverse: Replication Data for: Antenatal care providers’ attitudes and beliefs towards maternal vaccination in Kenya. https://doi.org/10.7910/DVN/43PPDD ^13^

The project contains the following extended data:

- KAB surveys.docx (Surveys in English and other languages)

## Disclaimer

The findings and conclusions in this report are those of the authors and do not necessarily represent the official position of the Centers for Disease Control and Prevention.
