# Supplementary material for: Antenatal care providers’ attitudes and beliefs towards maternal vaccination in Kenya
Source: Gates Open Res. 2021 Apr 22;4:19. Originally published 2020 Feb 5. [Version 2] doi: 10.12688/gatesopenres.13091.2 (PMC8181780; doi:10.12688/gatesopenres.13091.2)
Supplement: Peer review history

# Peer review: Reviewer response for version 2

**DOI:** 10.21956/gatesopenres.14423.r30606

**Published:** 2021-06-04

**Authors:** Zhihua Liu

**Affiliations:** 1 Department of Gynaecology, Affiliated Shenzhen Maternity and Child Healthcare Hospital, Southern Medical University, Shenzhen, China

I should have the two answers on the following from the authors:

Results:

1. Question 1:
*"Providers responded that **myths and misconceptions about vaccines in the society did not affect their decisions related to maternal vaccination**. A majority also expressed that political leaders do not influence provider’s decision to accept vaccines. Similarly, most participants disagreed that ethnic/cultural background or religious beliefs influenced their attitudes or beliefs towards vaccination. ( Table 3)."*
  But, in Table 3 I found that the results have some conflict. I have marked that in Table 3 - please see the attachment here.
2. Question 2:
*"...Furthermore, **78% believed** that pregnant women take all the scheduled vaccines even when they migrate to new places. In addition, they feel that their patients trusted their suggestions and information about vaccine recommendation."*
  Please clarify, who believed?

Please correct, modify, and then consider publishing.

Is the work clearly and accurately presented and does it cite the current literature?

Partly

If applicable, is the statistical analysis and its interpretation appropriate?

Not applicable

Are all the source data underlying the results available to ensure full reproducibility?

No

Is the study design appropriate and is the work technically sound?

Partly

Are the conclusions drawn adequately supported by the results?

No

Are sufficient details of methods and analysis provided to allow replication by others?

Partly

Reviewer Expertise:

Gynecologic-oncology

I confirm that I have read this submission and believe that I have an appropriate level of expertise to confirm that it is of an acceptable scientific standard.

# Peer review: Reviewer response for version 1

**DOI:** 10.21956/gatesopenres.14240.r29859

**Published:** 2020-11-18

**Authors:** Zhihua Liu

**Affiliations:** 1 Department of Gynaecology, Affiliated Shenzhen Maternity and Child Healthcare Hospital, Southern Medical University, Shenzhen, China

We need this type study for the Vaccination strategies very much. But this paper can not answer any of my concerns about the Vaccination strategies. The study design is not appropriate; about the 'most of those surveyed were female nurses of Luo ethnicity', 'we did not collect data on validity or reliability.‘ ’we did not collect information on the number of providers that were approached and declined to participate.'

 Table 1. ***Healthcare Staff: ***Just have 1 ***ob/gyn ***doctor. We need some more discussion about the inclusion criteria.

 Table 3: The data has some conflicts with the writing description.

 Table 4: Maybe not focusing on the main topic or key concerns. In my opinion, it should be deleted.

 All of the above let me cannot believe the results and conclusion.

Is the work clearly and accurately presented and does it cite the current literature?

Partly

If applicable, is the statistical analysis and its interpretation appropriate?

Not applicable

Are all the source data underlying the results available to ensure full reproducibility?

No

Is the study design appropriate and is the work technically sound?

Partly

Are the conclusions drawn adequately supported by the results?

No

Are sufficient details of methods and analysis provided to allow replication by others?

Partly

Reviewer Expertise:

Gynecologic-oncology

I confirm that I have read this submission and believe that I have an appropriate level of expertise to state that I do not consider it to be of an acceptable scientific standard, for reasons outlined above.

# Response

**Published:** 2020-11-19

**Authors:** Ines Gonzalez Casanova

Thank you for your review. We have edited the article to add the inclusion criteria. The small number of physicians and single ob/gyn is a reflection of the reality in Kenya where most ANC services are provided by nurses. We agree that this study does not necessarily represent the attitudes and beliefs of all ANC providers in Kenya, however it does provide valuable information from a large sample of providers from four different geographic regions of this country. 

 The questionnaires and the data for replication are available in the following links:

 Underlying data

 Harvard Dataverse: Replication Data for: Antenatal care providers’ attitudes and beliefs towards maternal vaccination in Kenya. https://doi.org/10.7910/DVN/43PPDD ^13^

 This project contains the following underlying data:

- MVAC_ANC_GATES manuscript 12 3 2019.tab (Survey responses and data dictionary)

Extended data

 Harvard Dataverse: Replication Data for: Antenatal care providers’ attitudes and beliefs towards maternal vaccination in Kenya. https://doi.org/10.7910/DVN/43PPDD ^13^

 The project contains the following extended data:

- KAB surveys.docx (Surveys in English and other languages)

# Peer review: Reviewer response for version 1

**DOI:** 10.21956/gatesopenres.14240.r29369

**Published:** 2020-08-18

**Authors:** Clarissa Simas

**Affiliations:** 1 Department of Infectious Disease Epidemiology, London School of Hygiene and Tropical Medicine, London, UK

This is a well written, important research into healthcare professional's attitudes towards maternal immunisation in Kenya. I am adding below my suggestions to the authors.

**Introduction:**

- Paragraph 3 - the following statement needs to be referenced: “most of the research assessing the knowledge, attitudes, and beliefs of health providers towards maternal immunization has been conducted in high-income settings.”
- Paragraph 3 - What are the authors referring as ‘ **characteristics**’? Demographic? Socioeconomic?: “The objective of this study was to assess attitudes, beliefs and **characteristics** of antenatal care providers towards maternal vaccination in Kenya." 

 

**Methods:**

- Paragraph 1 - if the larger study this is part of has been published elsewhere, I suggest referencing in the methods section of this paper.
- Data collection - has the KAB instrument been validated for use in this population? Please provide further information.

 

**Discussion:**

- Paragraph 1: Perhaps I misunderstood what the authors mean, but it is not clear to me what the ‘avenues to improve coverage’ identified in this study are. A key result of this study is that ANC providers hold a high positive regard for maternal immunization while coverage remains low, indicating that coverage issues are likely not due to vaccine hesitancy among ANC providers. Hence it is not clear to me what the ‘avenues for improving coverage’ suggested by the authors are.

Is the work clearly and accurately presented and does it cite the current literature?

Yes

If applicable, is the statistical analysis and its interpretation appropriate?

I cannot comment. A qualified statistician is required.

Are all the source data underlying the results available to ensure full reproducibility?

No source data required

Is the study design appropriate and is the work technically sound?

Yes

Are the conclusions drawn adequately supported by the results?

Partly

Are sufficient details of methods and analysis provided to allow replication by others?

Yes

Reviewer Expertise:

Qualitative research, acceptance of maternal vaccines in low and middle income countries, vaccine confidence and hesitancy.

I confirm that I have read this submission and believe that I have an appropriate level of expertise to confirm that it is of an acceptable scientific standard.

# Response

**Published:** 2020-11-18

**Authors:** Ines Gonzalez Casanova

Thank you for your valuable feedback. We have edited the manuscript to incorporate your suggestions. Specifically, we added citations in the introduction and methods, and clarified the confusing statements in the introduction, methods and discussion.
